# Supplementary material for: Comparative Multi-Omics Analysis Reveals Lignin Accumulation Affects Peanut Pod Size
Source: Int J Mol Sci. 2022 Nov 4;23(21):13533. doi: 10.3390/ijms232113533 (PMC9658497; doi:10.3390/ijms232113533)
Supplement: Supplementary file 1 [file ijms-23-13533-s001.zip › Figures S1-S4.pdf]

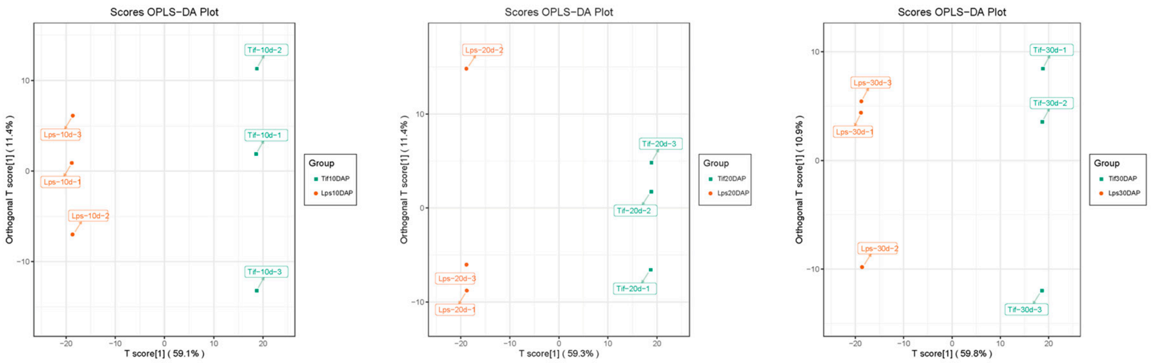

Figure S1. OPLS-DA plot

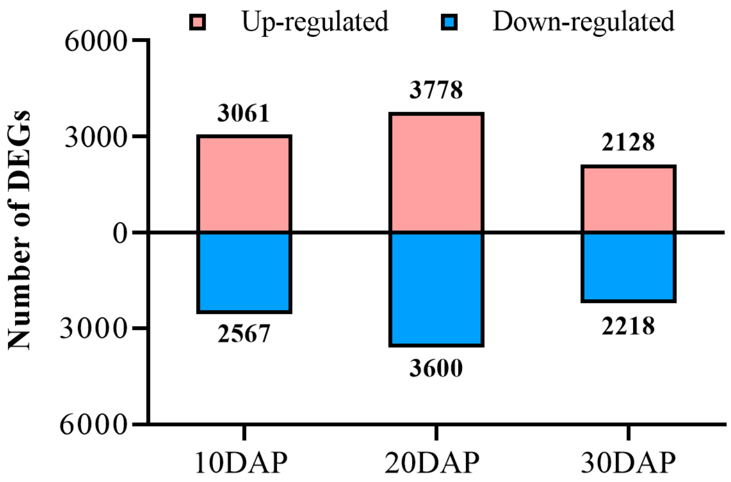

Figure S2. Number of DEGs

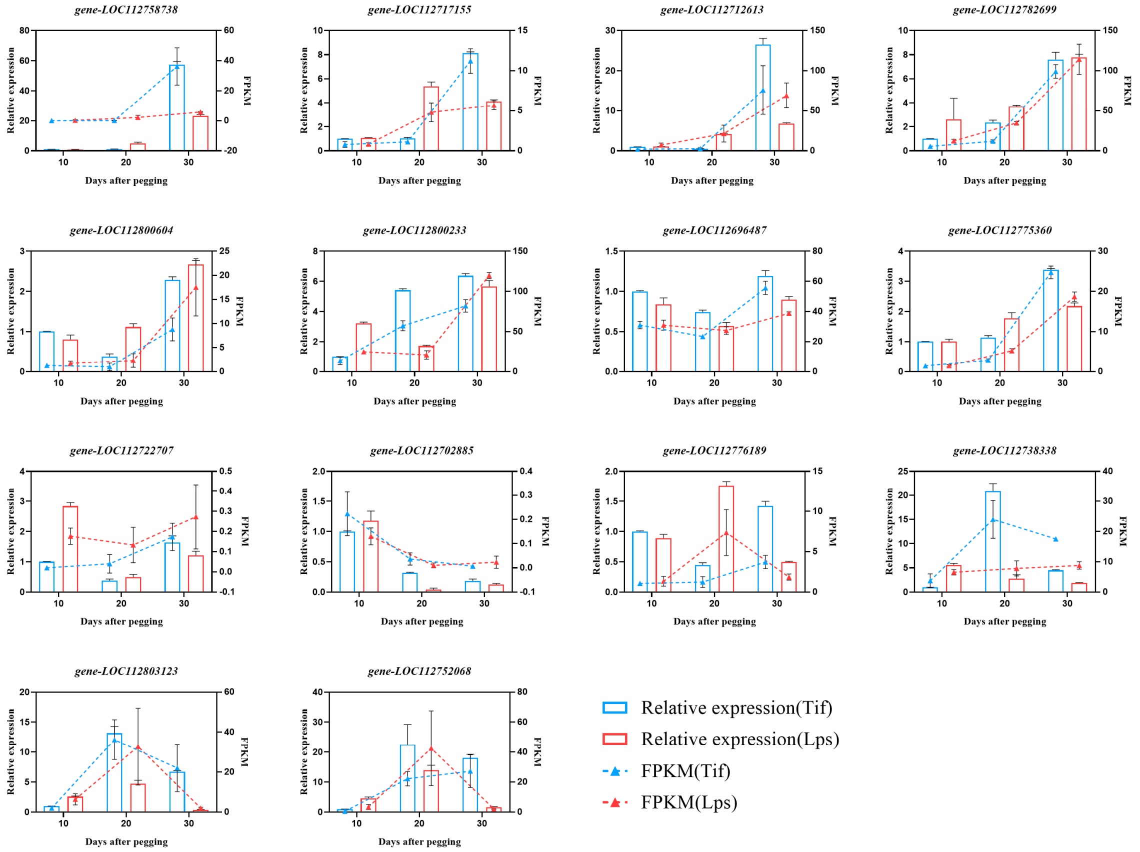

Figure S3. qRT-PCR Analysis

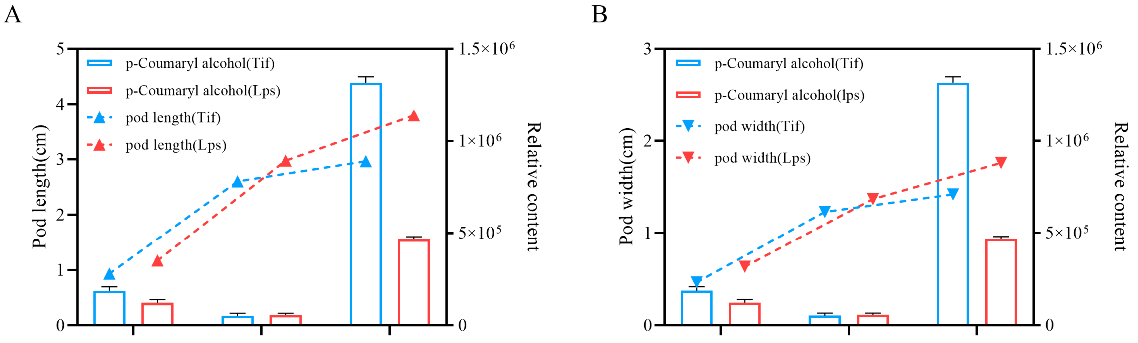

Figure S4. Dynamic changes of p-coumaryl alcohol content and pod length (A) and width (B)
